# Hierarchical Composites with Electrophoretically Deposited Carbon Nanotubes for In Situ Sensing of Deformation and Damage

**DOI:** 10.3390/nano10071262

**Published:** 2020-06-28

**Authors:** Colleen M. Murray, Sagar M. Doshi, Dae Han Sung, Erik T. Thostenson

**Affiliations:** 1 Center for Composite Materials, University of Delaware, 101 Academy Street, Newark, DE 19716, USA; cmmurray@udel.edu (C.M.M.); smdoshi@udel.edu (S.M.D.); dhsung@udel.edu (D.H.S.); 2 Department of Materials Science and Engineering, University of Delaware, 101 Academy Street, Newark, DE 19716, USA; 3 Department of Mechanical Engineering, University of Delaware, 101 Academy Street, Newark, DE 19716, USA

**Keywords:** glass-fiber composites, cross-ply laminates, crack density, structural health monitoring, piezoresistive sensors

## Abstract

As composites are used increasingly in structural components, novel techniques for detecting micro-scale damage are required. Their nanoscale size and high aspect ratio allow carbon nanotubes to create electrically conductive pathways that enable sensing. In this work, carbon nanotubes are deposited onto glass fabric using electrophoretic deposition to create hierarchical composites. Polyethylenimine functionalized carbon nanotubes are deposited from an aqueous dispersion using an electric field. Symmetric cross-ply composites are investigated as a model system to demonstrate the ability to detect incipient damage and transverse microcracks. The specimens are subjected to tensile loading, and a resistance increase is observed because of two key mechanisms—A reversible change in nanotube-nanotube tunneling gaps due to elastic straining of the network and a permanent severing of paths in the conducting network due to formation of transverse cracks in the 90° plies. By analyzing the electrical response, the damage state can be identified. Acoustic emission sensors are used to validate the results. The strength and Young’s modulus of the composites with integrated carbon nanotubes are similar to the control specimens. Crack density measurements using edge replication reveal that transverse cracking can be suppressed, demonstrating multi-functionality with improved damage tolerance and integrated sensing.

## 1. Introduction

Increasing use of fiber-reinforced polymer composites in critical load bearing and structural applications in recent years has necessitated the development of innovative techniques for damage sensing. The formation of microcracks at strains lower than the failure strain could potentially have a significant effect on the mechanical properties, thereby affecting the long-term durability and performance standards. Decrease in the elastic modulus due to the accumulation of microcracks may be used as an indicator of the amount of damage [1,2]. However, the changes in the elastic modulus are very small, as the elastic properties are dominated by the fiber reinforcement. Conventional sensors, such as strain gauges, have been used by researchers for sensing in composites, but they are quasi-point sensors and can detect damage that is in the immediate vicinity only. While fiber optic sensors are able to sense in large areas, their use with pre-existing structures is challenging and integrating them with fiber composites is difficult without altering the mechanical properties [3].

Due to their remarkable mechanical and electrical properties, carbon nanotubes are excellent candidates for in situ distributive sensing of strain and damage in composites. Carbon nanotubes have a fiber-like structure and a very high aspect ratio, along with good electrical conductivity. Therefore, carbon nanotubes can be integrated with fiber reinforced composites without significantly compromising the mechanical properties to create an electrically conductive percolating network. The conductive network is extremely sensitive to strain due to the change in contact resistance between the nanotubes, and the formation of microcracks disrupt the conductive electrical network formed by the carbon nanotubes [4,5,6]. The ability to detect micron-sized crack is unique to carbon nanotube-based composites, as a nanoscale conductor is required to detect micron-sized crack. 

Researchers have demonstrated improvement in the electrical conductivity at small concentrations of carbon nanotubes. Typically, carbon nanotubes are mixed in the resin (matrix) and dispersed using a variety of techniques such as shear mixing, mechanical stirring, surfactants or three-roll milling. Gojny et al. [7] and Thostenson and Chou [6] were among the first ones to disperse carbon nanotubes using a three-roll mill. This technique offers significant advantages such as maintaining the aspect ratio of the nanotubes and the ability to disperse nanotubes without the use of solvents. Researchers have also investigated the effect of functionalization of carbon nanotubes on dispersion effectiveness and mechanical properties of composites [8,9]. However, adding carbon nanotubes significantly affects the viscosity of the resin and filtering of nanotubes may be a challenge when fabricating larger parts.

Therefore, researchers have explored direct hybridization techniques such as chemical vapor deposition (CVD) [10,11], spray coating [12,13], and dip coating [14,15,16]. Thostenson et al. [10] studied the influence of carbon nanotubes grown on carbon fibers using carbon vapor deposition. The selective reinforcements allowed for improvement of the fiber-matrix interphase. However, CVD is energy intensive and may degrade the substrate fibers due to the high processing temperatures. Zhang and coworkers [12,13] utilized a spray coating technique to disperse carbon nanotubes into carbon and glass fiber composites to enable sensing capabilities. Dip coating has been used by researchers to deposit carbon nanotubes on non-woven fabrics to create sensing skins for strain sensing, monitoring fatigue cracks, and damage detection in joints [14,15,16]. While spray coating and dip coating are facile and cost effective, they do not offer the tailorability and control over the deposition of carbon nanotubes. It is challenging to control the nanotube coating thickness on all the fibers within the tows.

In this study, an electrophoretic deposition (EPD) technique was used for deposition of functionalized carbon nanotubes. An aqueous dispersion of carbon nanotubes functionalized with polyethyleneimine was created and a direct current (DC) electric field was used for the deposition on unidirectional glass fibers. The use of EPD for deposition of carbon nanotubes on nonconductive substrates has been demonstrated in our prior research [17,18,19]. Cross-ply composites [0/90]_s_ were manufactured and tested under monotonic and cyclic tensile loads for growth and accumulation of transverse microcracks. The control and carbon nanotube specimens have comparable strength and elastic modulus. The carbon nanotube coating enables strain sensing as well as tracking of the evolution of damage. By analyzing the hysteresis behavior of the electrical resistance response, the resistance change due to the crack formation/crack re-opening, elastic deformation, and damage was examined to obtain a deeper understanding of the damage evolution process.

## 2. Materials and Methods 

### 2.1. Materials

Unidirectional E-glass fabric (Style 7721, Thayercraft Inc., High Point, NC, USA) with a silane sizing was used with epoxy resin (EPON 862, Hexion, Columbus, OH, USA) cured using an aromatic diamine curing agent (EPI-KURE W, Hexion, Columbus, OH, USA). The nanoscale reinforcement was multi-walled carbon nanotubes (MWCNTs) with diameters on the order of 15–20 nm and lengths over 10 μm (CM95, Hanwha Nanotech, Seoul, South Korea). In order to use these nanotubes in the deposition process, they were functionalized with polyethylenimine (PEI) (M_w_ = 25,000, branched, Sigma–Aldrich, St. Louis, MO, USA) using an ultrasonication-ozonolysis process. Polyethylenimine functionalization is used to create a stable dispersion and assist in the mobility of the nanotubes.

### 2.2. Functionalization and Electrophoretic Deposition

A 1 g/L aqueous dispersion of carbon nanotubes was prepared by mixing MWCNTs in ultra-pure water (18.2 MΩ). The mixture was pumped through a wand sonicator (Sonicator 3000, Misonix, Farmingdale, NY, USA) while ozone gas was introduced using an oxygen concentrator (OxyMax 8, Longevity Resources, Sidney, BC, Canada) and a corona-discharge ozone generator (EXT 120-T, Longevity Resources, Sidney, BC, Canada). The sonicator was programmed with a duty cycle, where the sonicator was on for 15 s and off for 10 s. The dispersion was exposed to the sonication and ozone treatment for 16 h. Once completed, 1 g of PEI was introduced for each gram of nanotubes before sonicating an additional 4 h. Glacial acetic acid was used to adjust the pH of the dispersion to ~6 to promote the protonation of amine groups in the PEI. 

The stable dispersion of PEI functionalized carbon nanotubes (PEI-MWCNTs) enables motion under an applied electric field. This phenomenon is called electrophoresis. In this study, a DC electric field was used to deposit carbon nanotubes. The glass fabric was placed in direct contact with the cathode using elastic bands to ensure uniform contact. The electrodes were placed parallel to each other, uniformly separated using electrically insulating spacers and submerged in the aqueous nanotube dispersion. An electric field strength of 17 V/cm was applied for 5 min. Four depositions were completed to evenly coat the fibers. Four plies were dried together under a vacuum bag 60 °C for 3 h to ensure a constant ply thickness.

### 2.3. Composites Manufacturing

Vacuum-assisted resin transfer molding (VARTM) was used to manufacture two types of laminates: (i) control specimens without nanotubes, and (ii) MWCNT-coated specimens. Four plies of glass fiber were placed on the tooling in a cross-ply [0/90]_s_ configuration. Peel-ply was placed onto the preform, followed by a distribution media. Curing agent Epikure W was used with EPON 862 epoxy resin in the weight ratio of 26.4 g per 100 g. The matrix was degassed in a vacuum oven at 50 °C for 30 min before infusing into the glass fiber preform pre-heated to 60 °C. The laminate was then cured at 130 °C for 6 h. The laminates with MWCNTs were manufactured in the same manner; however, a 6 mm region was masked using high temperature tape prior to infusion for marking of electrodes for resistance measurements after curing. 

### 2.4. Mechanical, Electrical, Thermal, and Microscopic Characterization

Electrically insulating glass fiber composite end tabs were adhered to the laminate using an epoxy adhesive (Hysol EA 9309, HENKEL, Dusseldorf, Germany) for electrical isolation. Two sets of specimens were machined from the laminate: one set was machined into standard ASTM D3039 [20] tensile specimens for obtaining the laminate Young’s modulus and strength and a second set was machined to half of the ASTM specimen size to allow more specimens for damage accumulation studies. Specimens for damage accumulation studies were polished along one edge to take edge replicas during testing to map the damage state. Strain was measured using resistive gages (CEA-06-125UN-350, 350 Ω, (Vishay Intertechnology, Malvern, PA, USA). Electrodes were applied to the MWCNT specimens with conductive silver paint (SPI Supplies, West Chester, PA, USA) to measure electrical changes of the specimen during testing. The electrical leads were attached using a silver filled conductive epoxy (Epoxies, etc.). 

All mechanical testing was conducted using a screw-driven controlled displacement load frame (Instron 5567, Norwood, MA, USA). Two types of tests were conducted: quasi-static testing with monotonically increasing loads up to failure and progressively increasing cyclic loading, with the load being held constant at each incremental load to allow for edge replication measurements. The displacement rate for all tests was 2 mm/min. Electrical measurements during testing were conducted using a voltage–current meter (Keithley 6430, Cleveland, OH, USA) with a constant source voltage of 20 V. The strain, resistance, and load data were acquired using a customized LabView program. An acoustic emission (AE) sensor (R6A-60KHz General Purpose AE Sensor, Physical Acoustics, Princeton Junction, NJ, USA) was attached to each specimen prior to testing using a hot melt adhesive and acoustic data was acquired in real-time (AEwin™, Physical Acoustics, Princeton Junction, NJ, USA). 

Edge replication was conducted to obtain an image of the damage state during the holding periods of the cyclic tests. A cellulose acetate replica film (Thick Cellulose Replicating Tape, 125 μm, 25 mm wide; Ted Pella Inc., Redding CA, USA) was pressed against the polished edge of the specimen and softened with an acetate replicating solution (Replicating Solution; Ladd Research Industries, Williston, VT, USA). The specimen edge acts like a mold for the cellulose acetate. When the acetone from the replicating solution dries, the replication tape hardens, creating a replica of the edge of the specimen. The transverse cracks in the 90° plies of the specimen appear on the replication film, which is then mounted onto a glass microscope slide and imaged using a 3D laser scanning confocal microscope (Keyence, VK-X2000, Itasca, IL, USA).

The microstructure of the composite was imaged using scanning electron microscopy and scanning gallium ion microscopy (SGIM), (Auriga 60 CrossBeam, Zeiss, Oberkochen, Germany). An accelerating voltage of 3 kV was used for SEM and the voltage for SGIM was varied to achieve the desired contrast. Using SGIM allows for the identification of conductive regions. The positively charged gallium ions interact with the conductive and nonconductive surfaces. The interaction of Ga^+^ ions with the conductive nanotube coating leads to the emission of secondary electrons, making the conductive regions appear bright. The electrically insulating regions, such as the glass fiber and epoxy matrix, appear dark [21,22]. Specimens were also imaged using conventional scanning electron microscopy. To minimize charging, samples were sputter coated prior to imaging. A vacuum sputter coater (Denton Desk, Denton Vacuum LLC, Moorestown, NJ, USA) was used to apply a thin conductive layer prior to analyzing the sample. 

The fiber volume fraction was determined using thermogravimetric analysis (TGA). Circular disks with a diameter of 4 mm were punched from the laminate and tested in a TGA/DSC (Mettler-Toledo International, Inc., Columbus, OH, USA). Tests were conducted in both air and nitrogen using a heating rate of 2 °C/min from 25 °C to 800 °C.

## 3. Results and Discussion

### 3.1. Composite Microstructure

**Figure 1:**
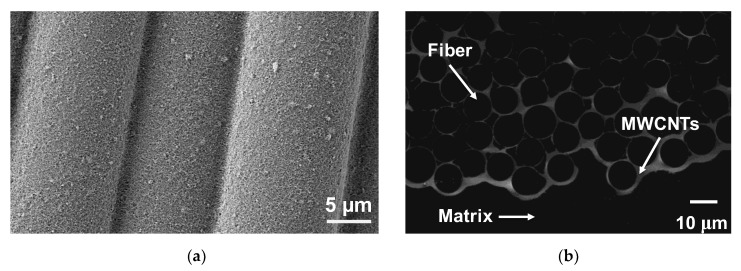
(**a**) SEM micrograph of PEI-MWCNTs deposited onto glass fibers and (**b**) SGIM micrograph of the composite cross-section showing the MWCNT coating (bright) penetrating the fiber bundle.

In order to analyze the carbon nanotube deposition on glass fabric, traditional SEM was used to view the morphology of the as-deposited nanotubes onto the fibers and SGIM was used to image the cross-sectional morphology and the integration of carbon nanotubes within the fiber bundles. Figure 1a shows an SEM image of glass fibers with MWCNTs deposited onto their surface via EPD. The functionalized nanotubes formed a uniform, porous PEI-MWCNT coating on the fiber surface. Figure 1b shows a SGIM image of the cross-section of the composite after infiltration with the epoxy matrix. Because the MWCNT coating was electrically conductive, it appeared as bright areas in the micrograph. It can be seen that the coating fully penetrated the E-glass fiber bundles. 

### 3.2. Mechanical and Thermal Characterization

**Figure 2:**
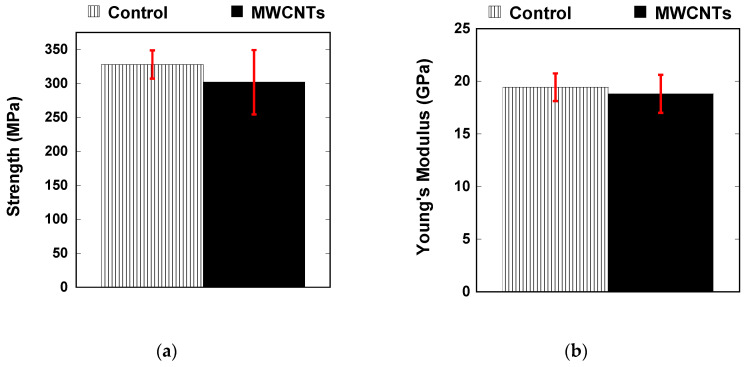
Mechanical properties of the laminates with and without carbon nanotubes showing (**a**) ultimate strength and (**b**) Young’s modulus for the control specimens and specimens with integrated MWCNTs.

Figure 2 shows the mechanical properties of the specimens with and without integrated carbon nanotubes. There was a slight reduction in the average strength (Figure 2a) of the laminate with integrated MWCNTs and slightly increased scatter, but both values are within experimental scatter. The Young’s modulus of the specimens also saw an increase in variation due to the presence of the carbon nanotubes; however, both types of samples have a modulus of about 19 GPa. For cross-ply laminates, the modulus was dominated by the 0° plies, where fibers were oriented in the loading direction. The carbon nanotubes were not expected to contribute much to this, because they are randomly oriented and concentrated at the fiber/matrix interface.

**Figure 3:**
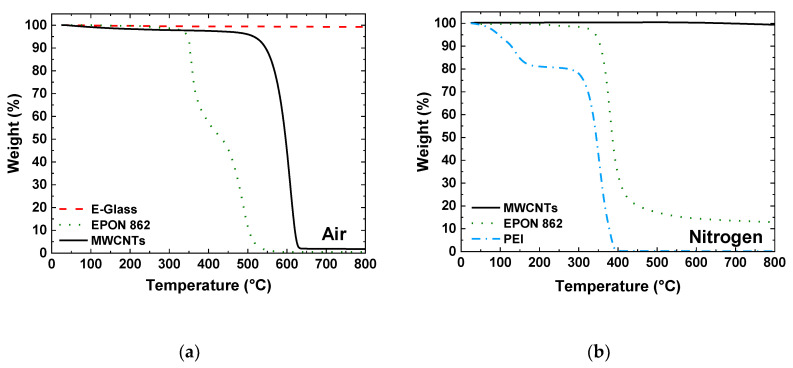
TGA thermographs of constituent materials in (**a**) air, showing decomposition of the epoxy and MWCNTs and minimal decomposition of the glass fiber, and (**b**) nitrogen, showing minimal decomposition of the MWCNTs, complete decomposition of the PEI and a residual mass of approximately 13% for the EPON 862 epoxy resin.

In order to determine the volume fraction of the constituents in the as-manufactured composite laminates, TGA was performed on the individual constituents as well as the control laminate and laminate with integrated MWCNTs. Figure 3a shows the TGA results in air of individual constituents. The E-glass did not show any significant change in mass over the temperature range of the test. The epoxy resin showed a two-step decomposition with an onset near 350 °C with no residual mass, indicating complete decomposition of the resin. For the MWCNTs, they begin to oxidize at temperatures above 500 °C and there is a very small residual mass, which is likely residual catalyst particles. In nitrogen (Figure 3b), the carbon nanotubes did not decompose while the PEI completely decomposed, leaving behind less than 0.5% residual mass. The epoxy did not burn off completely, likely due to the oxidation of the aromatic groups, and showed a residual mass of 13%. After the test, there was visible char remaining in the TGA pan.

**Figure 4:**
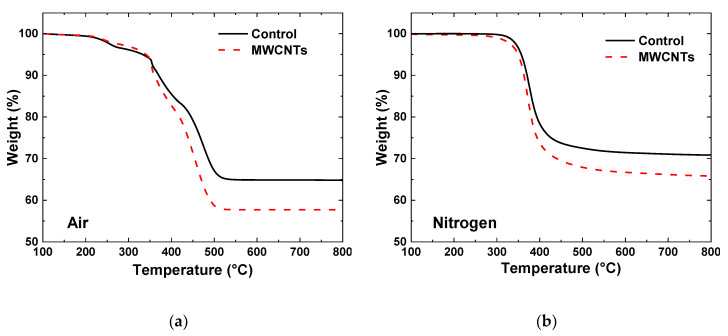
TGA thermographs for the control and MWCNT specimens tested in an (**a**) air and (**b**) nitrogen environments.

The TGA specimens cut from laminates are tested using a similar process. Figure 4a shows the TGA thermographs for both the control and MWCNT specimens in an air environment. Since the E-glass fibers do not decompose in air and the epoxy completely decomposes the residual mass of the control specimens is only glass fibers and show a residual mass of 65%. For specimens with integrated MWCNTs tested in air there is a lower overall residual mass. Since both the MWCNTs and epoxy degrade in air, the difference in the residual mass is due to the additional mass of carbon nanotubes in the laminate. Overall, the difference in the mass percentage was about 5%. The specimens that were tested in a nitrogen (Figure 4b) environment yielded a residual mass composed of epoxy char (~13% of the epoxy mass) along with the glass fiber and MWCNTs. As a result, the residual mass was significantly higher for the specimens tested in a nitrogen environment.

From these thermographs, the volume fractions were determined. The weight fraction of the two-part control system (i.e., fiber, matrix) can be calculated directly from the TGA thermograph in air (Figure 4a). Once the weight fractions were obtained, the density of the composite was calculated and used to determine the fiber volume fraction. These calculations were completed under the assumption of no voids, and the density of the fiber (*ρ*_f_) and matrix (*ρ*_m_) were 2.55 and 1.20 g/cm^3^, respectively. Table 1 summarizes the weight and volume fractions for the control laminate. 

In order to determine the three-part MWCNT system, it was assumed that the fiber volume fraction was the same for both laminates. This assumption was based on the observation that there was no thickness variation between the control and MWCNT specimens, and both specimens showed nearly identical Young’s modulus. The composite Young’s modulus was dominated by load-carrying fibers. The remaining volume fraction was shared between the volume fraction of the matrix and MWCNT. From TGA in air (Figure 4a) of the three-component MWCNT system, we can calculate the weight fraction of fibers since the matrix and MWCNTs degrade completely. Since neither the glass fibers nor MWCNTs decompose in nitrogen, the weight fraction of the matrix can be calculated from the residual mass of the three-component system in nitrogen accounting for 13% residual char of the matrix (Figure 4b). The CNT weight fraction can be calculated directly from the matrix and fiber weight fractions.

In order to estimate the volume fraction of the MWCNTs, the density must be known and is difficult to measure experimentally. Reported densities for MWCNTs are typically between 1.6–2.1 g/cm^3^. Table 2 summarizes the matrix volume and weight fraction for varying MWCNT density, and the concentration of MWCNTs is in the range of 3.7–4.8% by volume.

### 3.3. Damage Sensing under Monotonic Tensile Loading

The damage accumulation process in cross-ply laminates has been studied extensively. At low strain, the laminate behaves elastically. First-ply cracking occurs due to the initiation of damage in the 90° plies. This damage initiation typically occurs near the fiber/matrix interface due to the high stress concentrations resulting from the mismatch in Young’s modulus between the fiber and matrix. The cracks grow further and form transverse cracks across the ply. These transverse cracks continue to accumulate and eventually saturate to a uniform crack spacing due to the load transfer and shear lag. Stress concentrations due to the cracks occur at the 0/90° ply interfaces and can result in fiber fracture in the 0° ply and also delamination at the ply interface. The stress–strain relationship typically shows a bilinear response with a characteristic knee after which the slope of the stress–strain curve decreases with damage accumulation occurring in the 90° ply.

**Figure 5:**
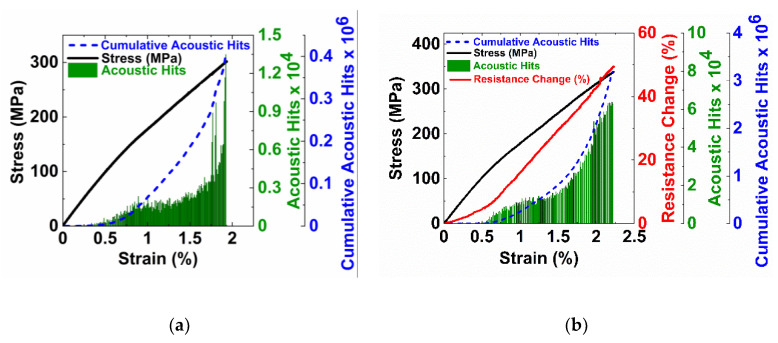
Monotonic testing was completed on the specimens both (**a**) without carbon nanotubes and (**b**) with carbon nanotubes. The stress, strain, and acoustic data was collected.

Figure 5a,b show the stress, strain, and acoustic emission activity of the control and MWCNT specimens, respectively. Both the control and MWCNT specimens showed the characteristic knee that occurs at about 0.6% strain. In Figure 5a, the control specimen shows very little acoustic activity prior to the characteristic knee, and there is no significant damage occurring and the laminate shows linear elastic behavior. Above the characteristic knee, there is a slight decrease in the slope of the stress–strain curve and a significant increase in acoustic activity likely due to the formation of transverse cracks in the 90° plies. The cumulative acoustic hits increase linearly in this region. At strains above 1.75%, there is a significant increase in acoustic hits, which likely corresponds to ply delamination as well as fiber breakage in the 0° plies, and an increase in the slope of the cumulative acoustic hits is observed.

**Figure 6:**
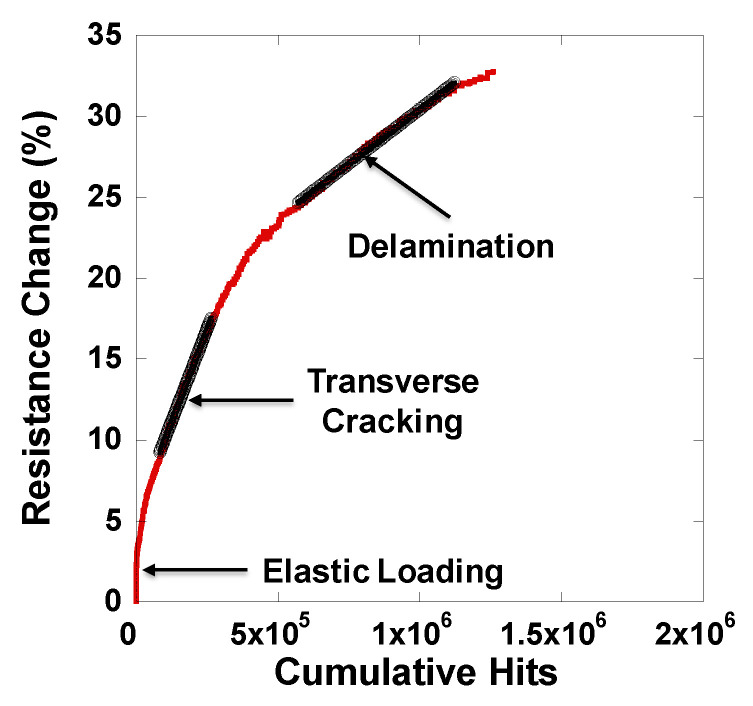
The electrical resistance change with cumulative acoustic hits showing the ability to identify the damage state in the composite laminate.

The stress–strain and acoustic emissions from the MWCNT specimen followed similar behavior as the control specimens. The sample showed no acoustic activity in the linear elastic region (0 to 0.6% strain). The change in electrical resistance is also measured for these specimens. The nature of the electrical resistance curve is similar to the stress–strain curve, linear until 0.6%, corresponding to the piezoresistivity of the specimen where the change in electrical resistance occurs due to the deformation of the nanotube network. The CNT-CNT tunneling resistance primarily influences the overall electrical resistance of the network, and as the network is strained in tension, the tunneling gaps increase, resulting in an overall change in electrical resistance. An increase in the slope of the resistance change curve is observed near 0.6% strain, which corresponds to the increase in acoustic activity and the characteristic knee in the stress–strain curve due to the formation of transverse cracks. The resistance continues to increase due to the continued transverse cracking. Figure 6 shows the resistance change and cumulative acoustic hits. Three distinct linear regions are observed. The first region shows the increase in electrical resistance at low strain with no acoustic activity. The second linear region in Figure 6 corresponds to the formation of transverse cracks. At these strains, the nanotube network is disrupted by the transverse microcracks, which will result in a significant increase in the resistance change and in the acoustic counts. The third region corresponds to strains at which we see ply delamination.

### 3.4. Damage Accumulation under Progressively Increasing Cyclic Loading

**Figure 7:**
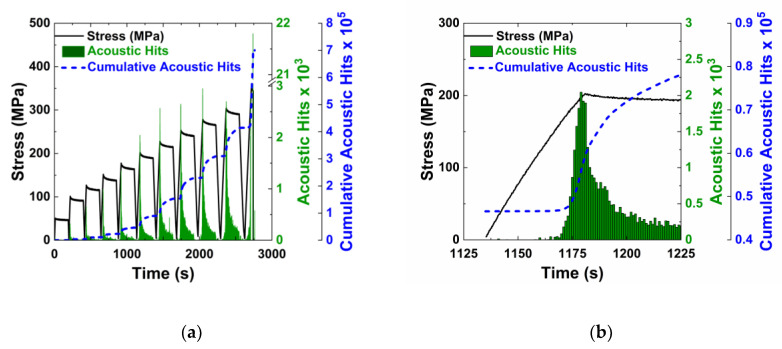
A control specimen without carbon nanotubes tested to failure under progressively increasing cyclic loading. (**a**) Stress and acoustic activity throughout the entire cyclic test, and (**b**) an examination of acoustic activity that occurs during the sixth loading cycle.

Figure 7 shows the transient stress curve and acoustic emission hits for the cyclic tests on the control specimen without integrated nanotubes. At the peak load for each cycle, the load was held constant for 3 min. For each cycle, new damage occurred only at stress levels higher than the peak stress of previous cycles. This is consistent with acoustic activity, an example of which is shown in Figure 7b, where increased acoustic activity is observed after the peak stress of the previous cycle. During the hold period, there were still some acoustic hits recorded. This behavior is characteristic of the well-known Kaiser Effect. The Kaiser Effect states that new damage does not occur until the previous stress has been surpassed. Once the previous peak stress of 175 MPa has been surpassed, new damage occurs as indicated by increased acoustic activity. 

**Figure 8:**
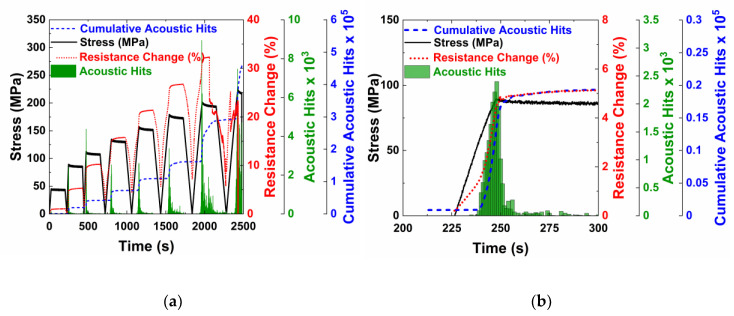
A specimen with carbon nanotubes under progressively increasing cyclic loading. (**a**) Stress, acoustic activity and electrical resistance throughout the cyclic test, and (**b**) an examination of the second loading cycle and the resistive response to the damage.

The characteristics that were seen in the control specimens can also be seen in the MWCNTs specimens (Figure 8a). The electrical resistance increases due to the increasing tunneling gap during the loading phase, is constant during the hold phase, and decreases in the unloading phase. At the end of first cycle, there is no permanent increase in the electrical resistance because the peak load was within the linear elastic range and no transverse cracks were formed. In subsequent cycles, a permanent change in electrical resistance is observed at the end of the cycle, likely due to the formation of microcracks, which sever the electrically conductive network.

**Figure 9:**
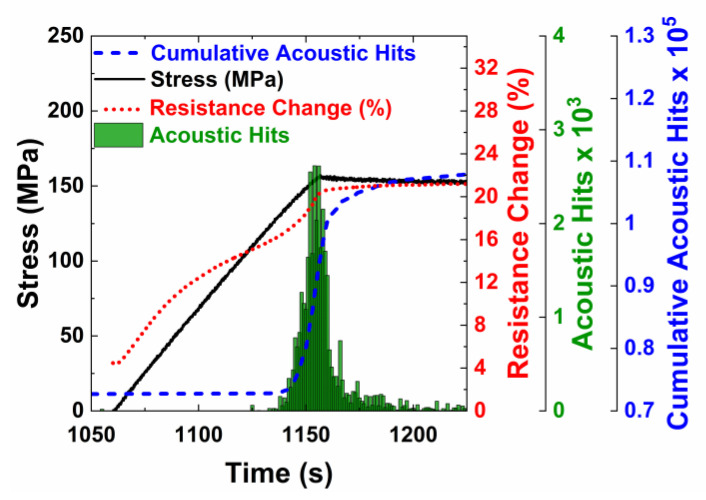
Stress, acoustic activity, and resistance change for the fifth loading cycle, where the non-linear electrical resistance response corresponds to crack re-opening, elastic loading, and new damage.

The samples with carbon nanotubes also demonstrate the Kaiser Effect, as shown by acoustic activity in load cycles higher than the second loading cycle. There is no acoustic activity recorded until the previous stress has been surpassed. Overall, the crack formation behavior is similar for the control and MWCNT specimens. Figure 8b shows the second loading cycle, where damage is first initiated. Like the MWCNT specimens undergoing monotonic loading (Figure 5b), there was a change in resistance during the initial loading due to the elastic piezoresistivity of the specimen. There was an abrupt change in the slope of the electrical resistance when acoustic activity was first observed, corresponding to the onset of transverse cracking in the specimen. The resistance versus strain response was linear when loaded within the elastic limit of the composites. But, for cycles with stress levels beyond this limit, the resistance response is non-linear, with three distinct sections as shown in Figure 9 for the fifth loading cycle. 

The three distinct phases were due to the transverse crack formation in the 90° plies, crack closing and re-opening due to the loading and unloading and the continued load bearing by the 0° plies. At the end of the unloading and before the loading of the new cycle began, the specimen was under no load and strain, and caused the microcracks to close and re-establish some of the electrical connections. During the initial stages of the subsequent loading cycle, the microcracks formed in previous cycles re-opened. The re-opening of the cracks caused a significant number of electrical contact points to break, leading to a higher slope of the resistance change curve. Once all of the previously formed cracks were re-opened due to the applied strain, the change in resistance in the next phase was due to piezoresistive elastic deformation and an increase in the tunneling gaps. The slope of this section was similar to the slope in the first cycle when the resistance change was only due to elastic piezoresistive loading. There was likely no formation of new microcracks as evidenced by no acoustic activity. In the next phase, an increase in the slope of the resistance change was observed, which was likely due to the formation and accumulation of new damage, which corresponds directly with increased acoustic activity.

**Figure 10:**
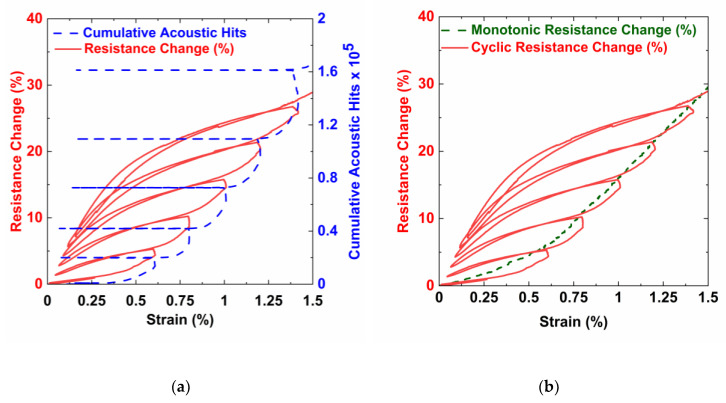
Graphs showing (**a**) resistance and accumulated acoustic hits with strain under cyclic loading and (**b**) comparison of the cyclic and monotonic resistance changes with strain.

Figure 10a shows the electrical resistance change and cumulative acoustic hits with strain for the loading/unloading cycles. As the specimen was cycled, the resistance was unable to return to zero because the piezoresistive network was permanently altered by the formation of cracks that severed the network, resulting in a permanent increase. As observed in the resistance response in Figure 9, the slope of resistance change in the initial part of the loading phase was higher due to the crack re-opening. The slope decreased, as the resistance change was only due to the piezoresistive deformation and increased towards the end of the loading phase due to the formation of the cracks. The Kaiser effect can also be clearly observed in the cumulative count graph. A horizontal line on the cumulative count graph means that no acoustic activity was in that phase. For all cycles, the cumulative count curve increases only towards the end of the loading cycle. Also, the increase in cumulative count initiates at around the peak strain of the previous cycle. There is very little acoustic activity during the hold period. Almost negligible acoustic hits were measured during the unloading phase, and as a result, the cumulative count curve is horizontal and overlaps with the early part of the loading phase of the next cycle. The damage states were able to be identified in both the cyclic and quasi-static tests. Figure 10b shows the quasi-static resistance curve follows the same trend as the cyclic resistance curve. This indicates that no additional damage was done to the network while determining the damage state of the specimens.

**Figure 11:**
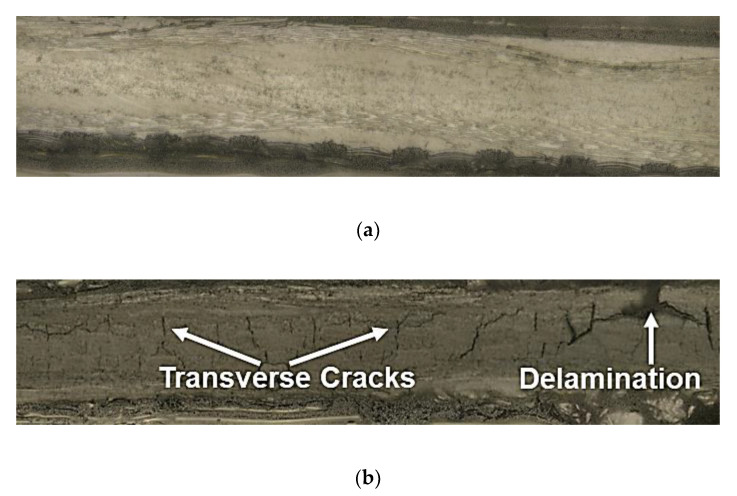
Micrographs showing damage states at varying loads (**a**) within the elastic region, no microcracks formed and (**b**) just before failure, transverse cracks and delamination can be observed.

In order to verify the damage state of the specimens, edge replicas were taken during the hold period of the cyclic tests. These replicas were analyzed using a confocal microscope to identify the microcracks that formed in the transverse layers of the laminates. Figure 11a,b shows the micrographs of the specimen cross-section captured at a load within the elastic region and just before failure, respectively. In Figure 11a, microcracks have not yet been formed, whereas in Figure 11b, uniformly spaced microcracks in the 90° plies as well as delamination along the interface of 0° and 90° plies, can be observed.

**Figure 12:**
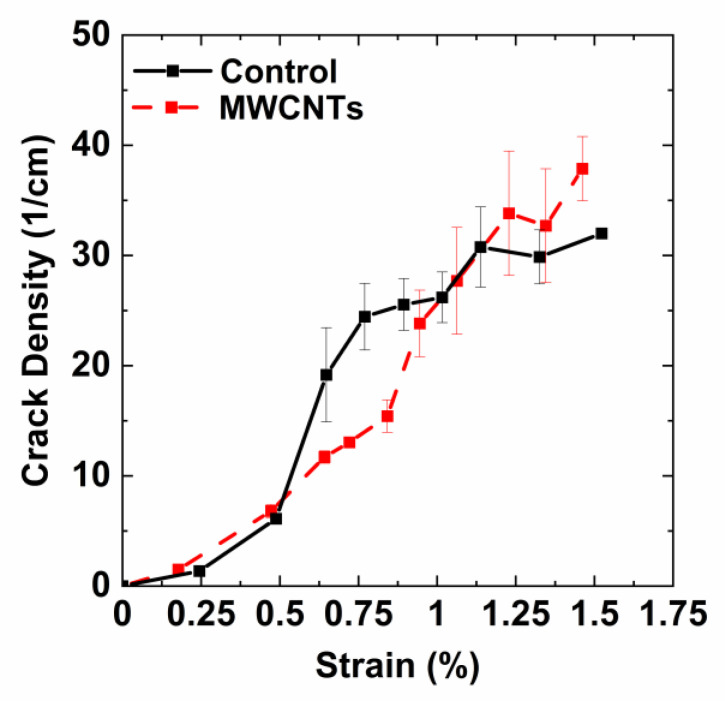
Measured crack density from edge replicas with increasing strain under cyclic loading for the control and MWCNT specimens.

The crack density was determined for the specimens during the hold periods. Figure 12 shows the average measured crack density curves for several specimens for the control and MWCNT specimens. The specimens with carbon nanotubes appeared to suppress crack formation in the initial stages of microcracking. For the control specimens, a sharper increase in crack density was observed when compared to the MWCNT specimens. At a strain of 0.75%, the crack density of the control specimens was 25 cracks/cm. At the same strain, the MWCNT specimen had a crack density of 12 cracks/cm. At 1.12% strain, the samples without nanotubes became saturated with a crack density of just over 30 cracks/cm. The MWCNT specimens become saturated with cracks at 1.25% strain with a crack density near 35 cracks/cm. The control specimens also reached a saturation crack density at a significantly lower strain (~1%) when compared to the MWCNT specimens (1.25%). Since cracking initiates at the fiber/matrix interface due to the stress concentration introduced by the mismatch in Young’s modulus between the fiber and matrix, these results suggest that the carbon nanotubes near the fiber/matrix interface may impede the crack growth by introducing a tough interphase around the fiber. For the MWCNT specimens, the cumulative acoustic hits are higher (Figure 5b), which may indicate that damage is forming, but the crack is arrested by the tough nanotube composite interphase.

## 4. Conclusions

With the increasing use of composites in structural applications, there is an increasing need to develop techniques that are capable of detecting micro-scale damage progression in composites, such as the initiation and propagation of transverse cracks and delamination. In this work, multi-walled carbon nanotube films are deposited onto unidirectional E-glass fabric using an industrially scalable EPD technique. Polyethylenimine functionalized carbon nanotubes are deposited from an aqueous dispersion to create hierarchically structured composites. Symmetric cross-ply composites were studied to establish the ability of the conductive nanotube network to detect incipient damage and the accumulation of transverse microcracks. Characterization results showed that the integration of carbon nanotubes had minimal influence on the ultimate strength and Young’s modulus as compared to the control specimens without nanotubes, which was expected because the in-plane fibers dominate the strength and stiffness of these laminates. The carbon nanotubes created a conductive, electrically percolating network that enabled in situ sensing in composites. The specimens were subjected to monotonic and cyclic tensile loading, and a resistance increase was observed due to the fact of two key mechanisms—A reversible resistance change due to the elastic straining of the carbon nanotube network and the permanent severing of conductive paths due to the formation of cracks. During testing, the results were validated using acoustic emission, with both laminates showing similar responses. Edge replication measurements reveal that transverse cracking can be suppressed. Cracking initiates at the fiber/matrix interface due to the stress concentrations arising from the mismatch in the fiber and matrix Young’s moduli. Carbon nanotubes near the fiber/matrix interface may impede the crack growth by introducing a tough interphase around the fiber. For the MWCNT specimens, the cumulative acoustic hits are also higher and may signify that cracks may be arrested by the tough nanotube composite interphase. The integration of MWCNTs in the composite shows their multi-functionality, where damage tolerance can be improved while adding integrated sensing functionality of the carbon nanotube network. 

## Figures and Tables

**Table 1:** The fiber and matrix volume fraction for the samples without carbon nanotubes.

	Fiber Volume Fraction	Matrix Volume Fraction
Average	0.466	0.534
Standard Deviation	0.0091	0.0091
Coefficient of Variation	1.96%	1.71%

**Table 2:** The matrix and nanotube volume fractions for samples based on a range of carbon nanotube densities.

Carbon Nanotube Density (g/cc)	Matrix Volume Fraction	Nanotube Volume Fraction
1.6	0.485	0.048
1.85	0.492	0.042
2.1	0.496	0.037
